# Role of MicroRNA in Osteoarthritis

**DOI:** 10.4172/2167-7921.1000239

**Published:** 2017-04-28

**Authors:** Mingcai Zhang, Kate Lygrisse, Jinxi Wang

**Affiliations:** 1 Harrington Laboratory for Molecular Orthopedics, Department of Orthopedic Surgery, University of Kansas Medical Center, Kansas City, Kansas, USA; 2 Department of Biochemistry & Molecular Biology, University of Kansas Medical Center, Kansas City, Kansas, USA

**Keywords:** MicroRNA, Osteoarthritis, Epigenetics, Gene expression, Biomarker

## Abstract

Although the potential effect of aberrant expression of catabolic and anabolic genes on the development of osteoarthritis (OA) is well-documented, the regulatory mechanism for the expression of these genes in articular chondrocytes remains to be elucidated. The recent advances in epigenetic studies have identified microRNA (miRNA) as one of the epigenetic mechanisms for the regulation of gene expression. This mini review highlights the role of miRNA in the regulation of gene expression in articular chondrocytes and its significance in the pathogenesis of OA, with a discussion on the potential of miRNA as a new biomarker and therapeutic target for OA. Further investigations are required to determine the specificity, sensitivity, and efficacy of miRNA for clinical applications.

## 1. Introduction

In contrast to genetics which is the study of hereditable variation in DNA sequences, epigenetics refers to the study of the changes in gene transcription activity caused by mechanisms other than changes in DNA sequences. Traditional epigenetic covalent modifications include DNA methylation and histone protein modifications (e.g. acetylation, methylation, phosphorylation, ubiquitination and sumoylation).

Recently, non-coding RNAs (ncRNAs) that possess epigenetic-like properties in the regulation of gene expression have also been considered as one of the epigenetic mechanisms [1,2]. With the use of high-throughput technologies, comprehensive assessment of the quantity of transcriptional molecules, including protein-coding messenger RNAs (mRNA) and ncRNAs, is now an area of rapid expansion in biomedical research of common diseases, such as Osteoarthritis (OA).

OA is the most common form of arthritis and is the leading cause of chronic disability in middle-aged and older populations [3]. Aberrant gene expression in articular chondrocytes of OA joints has been well documented in both animal and humans studies. However, the underlying regulatory mechanism that causes aberrant gene expression in OA cartilage has not yet been elucidated.

This review will first highlight the role of microRNA (miRNA), one of the most studied ncRNAs, in the regulation of aberrant gene expression in articular chondrocytes as it relates to the pathogenesis of OA, and then discuss the potential use of miRNA as a biomarker and potential therapeutic target for OA.

## 2. miRNA and OA

### 2.1 Biogenesis of miRNA

Classically, a gene is assumed to be transcribed into an mRNA and then translated into a protein; however, the discovery of genes encoding ncRNAs has extended the definition of a gene. The ncRNA genes produce transcripts functioning as structural, catalytic, or regulatory RNAs rather than being translated into proteins. Based on their length, ncRNAs can be divided into short ncRNAs (<30 nucleotides) and long ncRNAs (lncRNAs, >200 nucleotides). Short ncRNAs include miRNAs, short interfering RNAs (siRNAs), and piwi-interacting RNAs (piRNAs) [4]. MiRNAs are transcribed from miRNA genes as long primary transcripts (pri-miRNAs) characterized by a hairpin structure and are processed as pre-miRNAs (around 70-nucleotides long) in the nucleus. After being transported into the cytoplasm, pre-miRNAs are cleaved by Dicer and then matured into miRNA of 22–24 nucleotides [5].

### 2.2 Aberrant gene expression in OA cartilage

Adult articular cartilage is an avascular tissue in which chondrocytes are the only cellular component. Articular chondrocytes maintain the low-turnover of the extracellular matrix (ECM) by delicately regulating the expression of catabolic and anabolic genes. Progressive degradation of articular cartilage ECM is the major pathophysiological feature of OA.

Increased expression of catabolic genes and decreased expression of anabolic genes are usually observed in OA chondrocytes, which disrupt the metabolic balance in articular cartilage.

A number of catabolic genes have been proposed to be involved in the development of OA, including the genes encode: 1) Aggrecanases, such as ADAMTS (a disintegrin and metalloproteinase with thrombospondin motifs)-4 and -5, two major aggrecanases which have been shown to play important role in development of OA [6–9]; 2) Collagenases, particularly MMP (matrix metalloproteinase)-13, a major type II collagen (COL2A1)-degrading collagenase, which contributes to the initiation and progression of OA [10,11]; 3) Pro-inflammatory cytokines, such as IL (interleukin)-1β, IL-6, and TNF-α (tumor necrosis factor)[12,13]; 4) RunX2 (Runt- related transcription factor 2), which contributes to the pathogenesis of OA by promoting chondrocyte hypertrophy and matrix breakdown in articular cartilage. Runx2+/− mice exhibit decreased cartilage destruction and osteophyte formation, along with reduced expression of type X collagen and MMP-13, as compared with wild-type mice [14]. Upregulation of these catabolic genes contributes to the increased degradation of articular cartilage ECM.

A number of anabolic genes have been proposed to be involved in the structure and function of articular cartilage, including the genes encode: 1) Aggrecan, a major proteoglycan in articular cartilage [15,16]; decreased aggrecan expression is often evident in OA cartilage [17,18]. 2) Collagens, collagen type II is one of the major ECM components of the articular cartilage. Mice bearing a small deletion mutation in type II collagen gene developed OA-like lesions [19]. 3) SOX9 (SRY-Box 9), SOX9 is a master transcription factor for chondrogenesis during the development of the skeletal system, in cooperation with SOX5 and SOX6 [20,21].

Although mice with conditional postnatal deletion of Sox9 in chondroytes do not develop OA [22], later OA usually is associated with decreased SOX9 expression [23]. 4) NFAT1 (Nuclear Factor of Activated T-cells 1), which is a member of the NFAT transcription factor family originally identified as a regulator of the expression of cytokine genes during the immune response [24,25].

NFAT1 has recently been shown to play an important role in maintaining the permanent cartilage phenotype in adult mice. Nfat1 knockout (Nfat1−/−) mice exhibit normal skeletal development, but display over-expression of numerous matrix-degrading proteinases and pro-inflammatory cytokines, as well as loss of collagen-2 and aggrecan during the early stage of OA.

These initial changes are followed by articular chondrocyte clustering, formation of chondro-osteophytes, progressive articular surface destruction, formation of subchondral bone cysts, and exposure of thickened subchondral bone, all of which resemble human OA [26]. Down regulation of these anabolic genes contributes to the decreased ECM synthesis, impairing the repair ability of articular cartilage.

### 2.3 Regulation of gene expression in OA by miRNAs

The importance of epigenetic regulation of gene expression to the development of OA has recently been reported [27–29]. A number of miRNAs have been identified to be involved in the pathogenesis of OA in recent epigenetic studies. miRNAs may directly bind to catabolic and anabolic mRNAs to regulate their expression at a post-transcriptional level in cytoplasm with a complimentary sequence to induce cleavage and degradation, or block translation [30–32].

New findings indicate that the regulatory effect of miRNAs on the expression of catabolic and anabolic genes in OA may take place at upstream levels prior to their transcription. First, miRNAs target upstream signaling pathways or transcription factors. The activity of several signaling pathways, such as NF-kappaB pathway [33,34], Wnt/beta-Catenin pathway [35], SIRT1/p53 pathway [36] and SDF1/CXCR4 pathway [37], were found to be modulated by miRNAs in chondrocytes during the development of OA.

Moreover, miRNAs have also been reported to regulate transcription factor SOX9 in the development of OA [38,39]. Second, miRNAs target upstream epigenetic factors. Histone deacetylase-2 [40], -4 [41–43], and NAD-dependent deacetylase sirtuin-1 [44] have been found to be regulated by miRNAs in OA cartilage, indicating that the interaction among different epigenetic mechanisms is involved in OA pathogenesis.

### 2.4 miRNA and treatment of OA

The development of disease-modifying pharmacologic therapy for OA currently faces major obstacles largely because the pathogenesis of OA remains unclear. The aberrant expression catabolic and anabolic genes is a well-characterized molecular finding in OA; however, clinical trials targeting a single inflammatory mediator or proteinase did not slow the progression of OA [45–47].

This is probably due to the involvement of multiple factors in the pathogenesis of OA. In this regard, upstream molecular regulators would be more favorable therapeutic targets.

MiRNAs could be potential upstream targets for treatment of OA as one miRNA may regulate several genes. Furthermore, miRNAs regulate gene expression in OA cartilage at multiple levels and in a sequence-specific manner [48,49]. However, a large number of miRNAs have recently been identified in OA joint tissues, and one gene may be regulated by several miRNAs (Table 1).

Further investigations are needed to identify the articular cartilage specific miRNA(s) and to validate their efficacy in animal models of OA and in patients with OA. Specific transcription factors that regulate multiple catabolic and anabolic genes, such as NFAT1 [26,27,29], could also be potential upstream targets for treatment of OA.

### 2.5 miRNA and OA biomarker

Currently, X-ray and MRI (magnetic resonance imaging) are the established methods for the diagnosis of OA in clinical practice. However, specific blood testing that can be used to aid in the diagnosis and monitoring of OA progression is still under development. Clinicians and scientists are striving for a novel molecule(s) which can be used as a biomarker for early OA detection and for monitoring the progression of OA [50].

Given the high frequency of miRNAs expression in OA and the remarkably stable form of miRNAs present in clinical samples of plasma and serum [51,52], miRNAs could be ideal blood-based biomarkers for OA [53].

However, more studies are needed to identify the OA-specific miRNAs with high sensitivity to OA changes.

## 3. Conclusion

The recent advances in epigenetic studies have shed light on the importance of miRNAs in regulation of gene expression at multiple levels related to the pathogenesis of OA. This warrants the potential of miRNAs as therapeutic targets for OA. The tissue-specificity and high frequency of miRNA expression in OA renders miRNAs novel molecules as potential biomarkers for diagnosing OA, monitoring OA progression, and evaluating treatment efficacy. Further studies are required to identify which miRNAs out of the large number of miRNAs reported in the literature (Table 1) have high specificity, sensitivity and efficacy and could be used for clinical validation in OA patients.

## Figures and Tables

**Table 1:** Summary of differentially expressed miRNAs and their target(s) in OA cartilage

miRNA	Species	Change in OA	Target gene	Reference
miR-125b	H	↓	ADAMTS4	[54]
miR-140	M	↑	ADAMTS5	[31,55,36]
Has-miR-15a	H	↓	ADAMTS5	[31,55,36]
miR-30a	H	↓	ADAMTS5	[31,55,36]
miR-98	R	↑	Bcl2	[57]
miR-199a	H	↓	COX2	[58]
miR-210	R	↓	DR6	[34]
miR-221-3p	H	↓	Est-1	[59]
miR-138-5p	H	↑	FOXC1	[60]
miR-21	H	↑	GDF5	[61]
miR-92a-3p	H	↓	HADC2	[62]
miR-365	H	↑	HDAC4	[43]
miR-142-3p	M	↓	HMGB1	[63]
miR-140	H	↓	IGFBP-5	[64]
miR-27a	H	↓	IGFBP-5	[64]
miR-381a-3p	H	↑	IKBalpha	[65]
Has-miR26a-5p	H	↓	iNOS	[66]
miR-26a	H	↓	KPNA3	[60]
miR-26b	H	↓	KPNA3	[60]
miR-139	H	↑	MCPIP	[67]
miR-373	H	↓	MECP-2	[68]
miR-27a	H	↓	MMP-13	[30,64,69,70]
miR-27b	H	↓	MMP-13	[30,64,69,70]
miR-127-5p	H	↓	MMP-13	[30,64,69,70]
miR-320	H	↓	MMP-13	[30,64,69,70]
miR-9	H	↓	NF-kappaB1	[33]
miR-634	H	↓	PIK3R1	[71]
miR-221-3p	H	↓	SDF1	[37]
miR-370	H	↓	SHMT-2	[68]
miR-34a	H	↑	SIRT1	[36, 44]
miR-449q	H	↑	SIRT1	[36, 44]
miR-145	H	↓	SMAD3	[72]
miR-146a	R	↑	SMAD5	[73]
miR-101	R	↑	SOX9	[39,74]
miR-30a	H	↑	SOX9	[39,74]
miR-125b-5p	H	↑	SYVN1	[75]
miR-130A	R	↓	TNFα	[76]
miR-145	H	↓	TNFRSF11B	[77]
miR-562-5p	H	↓	TRAF2	[78]

HSA: Homo Sapiens; H: Human; M: Mouse; R: Rat↑: Upregulation; ↓: Down Regulation

## Abbreviations

OA: Osteoarthritis
MiRNA: MicroRNA
NcRNA: Non-coding RNA
MRNA: Messager; RNA
SiRNA: Short Interfering RNA
piRNA: Piwi-interacting RNA
ECM: Extracellular matrix
ADAMTS: A Disintegrin and Metalloproteinase with Thrombospondin Motifs
MMP13: Matrix Metalloproteinase 13
COL2: Type II Collagen
IL-1β: Interlukin 1-β
COL9: Type IX Collagen
TNF-α: Tumor Necrosis Factor-α
Runx2: Runt-Related Transcription Factor 2
NFAT: Nuclear Factor of Activated T-cells

## References

[1] Saetrom P, Snove O, Rossi JJ (2007). Epigenetics and microRNAs. Pediatr Res.

[2] Dawson MA, Kouzarides T (2012). Cancer epigenetics: from mechanism to therapy. Cell.

[3] Murphy L, Helmick CG (2012). The impact of osteoarthritis in the United States: a
population-health perspective. Am J Nurs.

[4] Mattick JS, Makunin IV (2006). Non-coding RNA. Hum Mol Genet.

[5] Ha M, Kim VN (2014). Regulation of microRNA biogenesis. Nature reviews Molecular cell biology.

[6] Tortorella MD, Malfait AM, Deccico C, Arner E (2001). The role of ADAM-TS4 (aggrecanase-1) and ADAM-TS5 (aggrecanase-2)
in a model of cartilage degradation. Osteoarthritis and cartilage/OARS, Osteoarthritis Research
Society.

[7] Glasson SS, Askew R, Sheppard B, Carito B, Blanchet T (2005). Deletion of active ADAMTS5 prevents cartilage degradation in a
murine model of osteoarthritis. Nature.

[8] Stanton H, Rogerson FM, East CJ, Golub SB, Lawlor KE (2005). ADAMTS5 is the major aggrecanase in mouse cartilage in vivo and
in vitro. Nature.

[9] Rogerson FM, Stanton H, East CJ, Golub SB, Tutolo L (2008). Evidence of a novel aggrecan-degrading activity in cartilage:
Studies of mice deficient in both ADAMTS-4 and ADAMTS-5. Arthritis and rheumatism.

[10] Little CB, Barai A, Burkhardt D, Smith SM, Fosang AJ (2009). Matrix metalloproteinase 13-deficient mice are resistant to
osteoarthritic cartilage erosion but not chondrocyte hypertrophy or
osteophyte development. Arthritis and rheumatism.

[11] Neuhold LA, Killar L, Zhao W, Sung ML, Warner L (2001). Postnatal expression in hyaline cartilage of constitutively
active human collagenase-3 (MMP-13) induces osteoarthritis in
mice. J Clin Invest.

[12] Goldring MB (2000). Osteoarthritis and cartilage: the role of
cytokines. Curr Rheumatol Rep.

[13] Kapoor M, Martel-Pelletier J, Lajeunesse D, Pelletier JP, Fahmi H (2011). Role of proinflammatory cytokines in the pathophysiology of
osteoarthritis. Nature reviews Rheumatology.

[14] Kamekura S, Kawasaki Y, Hoshi K, Shimoaka T, Chikuda H (2006). Contribution of runt-related transcription factor 2 to the
pathogenesis of osteoarthritis in mice after induction of knee joint
instability. Arthritis and rheumatism.

[15] Malfait AM, Liu RQ, Ijiri K, Komiya S, Tortorella MD (2002). Inhibition of ADAM-TS4 and ADAM-TS5 prevents aggrecan degradation
in osteoarthritic cartilage. The Journal of biological chemistry.

[16] Song RH, Tortorella MD, Malfait AM, Alston JT, Yang Z (2007). Aggrecan degradation in human articular cartilage explants is
mediated by both ADAMTS-4 and ADAMTS-5. Arthritis and rheumatism.

[17] Eid K, Thornhill TS, Glowacki J (2006). Chondrocyte gene expression in osteoarthritis: Correlation with
disease severity. Journal of orthopaedic research: official publication of the Orthopaedic
Research Society.

[18] Chambers MG, Kuffner T, Cowan SK, Cheah KS, Mason RM (2002). Expression of collagen and aggrecan genes in normal and
osteoarthritic murine knee joints. Osteoarthritis and cartilage/OARS, Osteoarthritis Research
Society.

[19] Saamanen AK, Salminen HJ, Dean PB, De Crombrugghe B, Vuorio EI (2000). Osteoarthritis-like lesions in transgenic mice harboring a small
deletion mutation in type II collagen gene. Osteoarthritis and cartilage/OARS, Osteoarthritis Research
Society.

[20] Lefebvre V, Li P, de Crombrugghe B (1998). A new long form of Sox5 (L-Sox5), Sox6 and Sox9 are coexpressed
in chondrogenesis and cooperatively activate the type II collagen
gene. Embo j.

[21] Bi W, Deng JM, Zhang Z, Behringer RR, de Crombrugghe B (1999). Sox9 is required for cartilage formation. Nat Genet.

[22] Henry SP, Liang S, Akdemir KC, de Crombrugghe B (2012). The postnatal role of Sox9 in cartilage. Journal of bone and mineral research: the official journal of the
American Society for Bone and Mineral Research.

[23] Lee JS, Im GI (2011). SOX trio decrease in the articular cartilage with the advancement
of osteoarthritis. Connect Tissue Res.

[24] Hodge MR, Ranger AM, Charles de la Brousse F, Hoey T, Grusby MJ (1996). Hyperproliferation and dysregulation of IL-4 expression in
NF-ATp-deficient mice. Immunity.

[25] Xanthoudakis S, Viola JP, Shaw KT, Luo C, Wallace JD (1996). An enhanced immune response in mice lacking the transcription
factor NFAT1. Science.

[26] Wang J, Gardner BM, Lu Q, Rodova M, Woodbury BG (2009). Transcription factor Nfat1 deficiency causes osteoarthritis
through dysfunction of adult articular chondrocytes. J Pathol.

[27] Rodova M, Lu Q, Li Y, Woodbury BG, Crist JD (2011). Nfat1 regulates adult articular chondrocyte function through its
age-dependent expression mediated by epigenetic histone
methylation. Journal of bone and mineral research: the official journal of the
American Society for Bone and Mineral Research.

[28] Zhang M, Egan B, Wang J (2015). Epigenetic mechanisms underlying the aberrant catabolic and
anabolic activities of osteoarthritic chondrocytes. The international journal of biochemistry & cell biology.

[29] Zhang M, Lu Q, Egan B, Zhong XB, Brandt K (2016). Epigenetically mediated spontaneous reduction of NFAT1 expression
causes imbalanced metabolic activities of articular chondrocytes in aged
mice. Osteoarthritis and cartilage.

[30] Meng F, Zhang Z, Chen W, Huang G, He A (2016). MicroRNA-320 regulates matrix metalloproteinase-13 expression in
chondrogenesis and interleukin-1beta-induced chondrocyte
responses. Osteoarthritis and cartilage/OARS, Osteoarthritis Research
Society.

[31] Lu X, Lin J, Jin J, Qian W, Weng X (2016). Hsa-miR-15a exerts protective effects against osteoarthritis by
targeting aggrecanase-2 (ADAMTS5) in human chondrocytes. International journal of molecular medicine.

[32] Wang G, Zhang Y, Zhao X, Meng C, Ma L (2015). MicroRNA-411 inhibited matrix metalloproteinase 13 expression in
human chondrocytes. American journal of translational research.

[33] Gu R, Liu N, Luo S, Huang W, Zha Z (2016). MicroRNA-9 regulates the development of knee osteoarthritis
through the NF-kappaB1 pathway in chondrocytes. Medicine.

[34] Zhang D, Cao X, Li J, Zhao G (2015). MiR-210 inhibits NF-kappaB signaling pathway by targeting DR6 in
osteoarthritis. Scientific reports.

[35] Cheleschi S, De Palma A, Pecorelli A, Pascarelli NA, Valacchi G (2017). Hydrostatic Pressure Regulates MicroRNA Expression Levels in
Osteoarthritic Chondrocyte Cultures via the Wnt/beta-Catenin
Pathway. International journal of molecular sciences.

[36] Yan S, Wang M, Zhao J, Zhang H, Zhou C (2016). MicroRNA-34a affects chondrocyte apoptosis and proliferation by
targeting the SIRT1/p53 signaling pathway during the pathogenesis of
osteoarthritis. International journal of molecular medicine.

[37] Zheng X, Zhao FC, Pang Y, Li DY, Yao SC (2017). Downregulation of miR-221-3p contributes to IL-1beta-induced
cartilage degradation by directly targeting the SDF1/CXCR4 signaling
pathway. Journal of molecular medicine.

[38] Martinez-Sanchez A, Dudek KA, Murphy CL (2012). Regulation of human chondrocyte function through direct
inhibition of cartilage master regulator SOX9 by microRNA-145
(miRNA-145). The Journal of biological chemistry.

[39] Chang T, Xie J, Li H, Li D, Liu P (2016). MicroRNA-30a promotes extracellular matrix degradation in
articular cartilage via downregulation of Sox9. Cell proliferation.

[40] Mao G, Zhang Z, Huang Z, Chen W, Huang G (2016). MicroRNA-92a-3p regulates the expression of cartilage-specific
genes by directly targeting histone deacetylase 2 in chondrogenesis and
degradation. Osteoarthritis and cartilage/OARS, Osteoarthritis Research
Society.

[41] Chen W, Sheng P, Huang Z, Meng F, Kang Y (2016). MicroRNA-381 Regulates Chondrocyte Hypertrophy by Inhibiting
Histone Deacetylase 4 Expression. International journal of molecular sciences.

[42] Song J, Jin EH, Kim D, Kim KY, Chun CH (2015). MicroRNA-222 regulates MMP-13 via targeting HDAC-4 during
osteoarthritis pathogenesis. BBA clinical.

[43] Yang X, Guan Y, Tian S, Wang Y, Sun K (2016). Mechanical and IL-1beta Responsive miR-365 contributes to
Osteoarthritis Development by Targeting Histone Deacetylase
4. International journal of molecular sciences.

[44] Park KW, Lee KM, Yoon DS, Park KH, Choi WJ (2016). Inhibition of microRNA-449a prevents IL-1beta-induced cartilage
destruction via SIRT1. Osteoarthritis and cartilage/OARS, Osteoarthritis Research
Society.

[45] Chevalier X, Goupille P, Beaulieu AD, Burch FX, Bensen WG (2009). Intraarticular injection of anakinra in osteoarthritis of the
knee: a multicenter, randomized, double-blind, placebo-controlled
study. Arthritis and rheumatism.

[46] Hellio le Graverand MP, Clemmer RS, Redifer P, Brunell RM, Hayes CW (2013). A 2-year randomised, double-blind, placebo-controlled,
multicentre study of oral selective iNOS inhibitor, cindunistat (SD-6010),
in patients with symptomatic osteoarthritis of the knee. Annals of the rheumatic diseases.

[47] Hellio Le Graverand-Gastineau MP (2009). OA clinical trials: current targets and trials for OA. Choosing
molecular targets: what have we learned and where we are
headed?. Osteoarthritis and cartilage/OARS, Osteoarthritis Research
Society.

[48] Shi J, Wei Y, Xia J, Wang S, Wu J (2015). MicroRNAs are potential prognostic and therapeutic targets in
diabetic osteoarthritis. J Bone Miner Metab.

[49] Wang H, Zhang H, Sun Q, Wang Y, Yang J (2017). Intra-articular Delivery of Antago-miR 483-5p Inhibits
Osteoarthritis by Modulating Matrilin 3 and Tissue Inhibitor of
Metalloproteinase 2. Molecular therapy: the journal of the American Society of Gene
Therapy.

[50] Li YH, Tavallaee G, Tokar T, Nakamura A, Sundararajan K (2016). Identification of synovial fluid microRNA signature in knee
osteoarthritis: differentiating early- and late-stage knee
osteoarthritis. Osteoarthritis and cartilage/OARS, Osteoarthritis Research
Society.

[51] Mitchell PS, Parkin RK, Kroh EM, Fritz BR, Wyman SK (2008). Circulating microRNAs as stable blood-based markers for cancer
detection. Proceedings of the National Academy of Sciences of the United States of
America.

[52] Beyer C, Zampetaki A, Lin NY, Kleyer A, Perricone C (2015). Signature of circulating microRNAs in
osteoarthritis. Annals of the rheumatic diseases.

[53] Bernard NJ (2014). Osteoarthritis: circulating miRNAs-early osteoarthritis
biomarkers?. Nature reviews Rheumatology.

[54] Matsukawa T, Sakai T, Yonezawa T, Hiraiwa H, Hamada T (2013). MicroRNA-125b regulates the expression of aggrecanase-1
(ADAMTS-4) in human osteoarthritic chondrocytes. Arthritis research & therapy.

[55] Miyaki S, Sato T, Inoue A, Otsuki S, Ito Y (2010). MicroRNA-140 plays dual roles in both cartilage development and
homeostasis. Genes & development.

[56] Ji Q, Xu X, Zhang Q, Kang L, Xu Y (2016). The IL-1beta/AP-1/miR-30a/ADAMTS-5 axis regulates cartilage
matrix degradation in human osteoarthritis. Journal of molecular medicine (Berlin, Germany).

[57] Wang J, Chen L, Jin S, Lin J, Zheng H (2016). MiR-98 promotes chondrocyte apoptosis by decreasing Bcl-2
expression in a rat model of osteoarthritis. Acta biochimica et biophysica Sinica.

[58] Akhtar N, Haqqi TM (2012). MicroRNA-199a regulates the expression of cyclooxygenase-2 in
human chondrocytes. Annals of the rheumatic diseases.

[59] Xu J, Liu Y, Deng M, Li J, Cai H (2016). MicroRNA221-3p modulates Ets-1 expression in synovial fibroblasts
from patients with osteoarthritis of temporomandibular joint. Osteoarthritis and cartilage/OARS, Osteoarthritis Research
Society.

[60] Yuan Y, Zhang GQ, Chai W, Ni M, Xu C (2016). Silencing of microRNA-138-5p promotes IL-1beta-induced cartilage
degradation in human chondrocytes by targeting FOXC1: miR-138 promotes
cartilage degradation. Bone & joint research.

[61] Zhang Y, Jia J, Yang S, Liu X, Ye S (2014). MicroRNA-21 controls the development of osteoarthritis by
targeting GDF-5 in chondrocytes. Experimental & molecular medicine.

[62] Mao G, Zhang Z, Huang Z, Chen W, Huang G (2017). MicroRNA-92a-3p regulates the expression of cartilage-specific
genes by directly targeting histone deacetylase 2 in chondrogenesis and
degradation. Osteoarthritis and cartilage/OARS, Osteoarthritis Research
Society.

[63] Wang X, Guo Y, Wang C, Yu H, Yu X (2016). MicroRNA-142-3p Inhibits Chondrocyte Apoptosis and Inflammation
in Osteoarthritis by Targeting HMGB1.

[64] Tardif G, Hum D, Pelletier JP, Duval N, Martel-Pelletier J (2009). Regulation of the IGFBP-5 and MMP-13 genes by the microRNAs
miR-140 and miR-27a in human osteoarthritic chondrocytes. BMC musculoskeletal disorders.

[65] Xia S, Yan K, Wang Y (2016). Increased miR-381a-3p Contributes to Osteoarthritis by Targeting
IkBalpha. Annals of clinical and laboratory science.

[66] Rasheed Z, Al-Shobaili HA, Rasheed N, Mahmood A, Khan MI (2016). MicroRNA-26a-5p regulates the expression of inducible nitric
oxide synthase via activation of NF-kappaB pathway in human osteoarthritis
chondrocytes. Archives of biochemistry and biophysics.

[67] Makki MS, Haqqi TM (2015). miR-139 modulates MCPIP1/IL-6 expression and induces apoptosis in
human OA chondrocytes. Experimental & molecular medicine.

[68] Song J, Kim D, Chun CH, Jin EJ (2015). miR-370 and miR-373 regulate the pathogenesis of osteoarthritis
by modulating onecarbon metabolism via SHMT-2 and MECP-2,
respectively. Aging cell.

[69] Akhtar N, Rasheed Z, Ramamurthy S, Anbazhagan AN, Voss FR (2010). MicroRNA-27b regulates the expression of matrix metalloproteinase
13 in human osteoarthritis chondrocytes. Arthritis and rheumatism.

[70] Park SJ, Cheon EJ, Lee MH, Kim HA (2013). MicroRNA-127-5p regulates matrix metalloproteinase 13 expression
and interleukin-1beta-induced catabolic effects in human
chondrocytes. Arthritis and rheumatism.

[71] Cui X, Wang S, Cai H, Lin Y, Zheng X (2016). Overexpression of microRNA-634 suppresses survival and matrix
synthesis of human osteoarthritis chondrocytes by targeting
PIK3R1. Scientific reports.

[72] Yang B, Kang X, Xing Y, Dou C, Kang F (2014). Effect of microRNA-145 on IL-1beta-induced cartilage degradation
in human chondrocytes. FEBS letters.

[73] Li J, Huang J, Dai L, Yu D, Chen Q (2012). miR-146a, an IL-1beta responsive miRNA, induces vascular
endothelial growth factor and chondrocyte apoptosis by targeting
Smad4. Arthritis research & therapy.

[74] Dai L, Zhang X, Hu X, Zhou C, Ao Y (2012). Silencing of microRNA-101 prevents IL-1beta-induced extracellular
matrix degradation in chondrocytes. Arthritis research & therapy.

[75] Ge FX, Li H, Yin X (2017). Upregulation of microRNA-125b-5p is involved in the pathogenesis
of osteoarthritis by downregulating SYVN1. Oncology reports.

[76] Li ZC, Han N, Li X, Li G, Liu YZ (2015). Decreased expression of microRNA-130a correlates with TNF-alpha
in the development of osteoarthritis. International journal of clinical and experimental pathology.

[77] Wang GD, Zhao XW, Zhang YG, Kong Y, Niu SS (2017). Effects of miR-145 on the inhibition of chondrocyte proliferation
and fibrosis by targeting TNFRSF11B in human osteoarthritis. Molecular medicine reports.

[78] Zhang G, Sun Y, Wang Y, Liu R, Bao Y (2016). MiR-502-5p inhibits IL-1beta-induced chondrocyte injury by
targeting TRAF2. Cellular immunology.

